# Vitamin A: A Key Inhibitor of Adipocyte Differentiation

**DOI:** 10.1155/2023/7405954

**Published:** 2023-02-01

**Authors:** Manal A. Malibary

**Affiliations:** Department of Food and Nutrition, Faculty of Human Sciences and Design, King Abdulaziz University, Jeddah, Saudi Arabia

## Abstract

Inhibiting adipocyte differentiation, the conversion of preadipocytes to mature functional adipocytes, might represent a new approach to treating obesity and related metabolic disorders. Peroxisome proliferator-activated receptor *γ* and CCAAT-enhancer-binding protein *α* are two master coregulators controlling adipogenesis both in culture and in vivo. Many recent studies have confirmed the relationship between retinoic acid (RA) and the conversion of embryonic stem cells into adipocytes; however, these studies have shown that RA potently blocks the differentiation of preadipocytes into mature adipocytes. Nevertheless, the functional role of RA in early tissue development and stem cell differentiation, including in adipose tissue, remains unclear. This study highlights transcription factors that block adipocyte differentiation and maintain preadipocyte status, focusing on those controlled by RA. However, some of these novel adipogenesis inhibitors have not been validated in vivo, and their mechanisms of action require further clarification.

## 1. Introduction

Obesity plays a central role in major health concerns, such as cardiovascular disease, autoimmune disease, and numerous metabolic abnormalities. Adipogenesis is the two-step process of transforming fibroblast-like progenitor cells into mature adipocytes. The first or commitment step is where fibroblast-like cells become preadipocytes, marked by increased expression of platelet-derived growth factor receptor (PDGFR)-*α* or -*β*. BOX2, without any morphological changes, is an example of a mesenchymal precursor. In the second step, preadipocytes differentiate into mature adipocytes [1].

The differentiation of preadipocyte cell lines is not well understood. However, preadipocyte cultures used to investigate cell line differentiation have shown that differentiation occurs in four main stages: the first stage is growth arrest, followed by mitotic clonal expansion (MCE), early differentiation, and terminal differentiation [2]. In addition, cells must proliferate before initiating the first differentiation stage; therefore, early differentiation markers are upregulated via cell–cell signalling [3].

The induction of the white adipocyte differentiation program is influenced by a comprehensive network of transcription factors and enhancers that function sequentially and collaboratively to activate adipocyte-specific gene expression and induce the adipocyte phenotype [4]. Chronological changes in key adipocyte marker genes during differentiation are the primary drivers for establishing the adipocyte phenotype. These changes are indicated by the accumulation of lipid droplets and the expression of early, intermediate, and late messenger RNA (mRNA)/protein markers [5] regulated at both transcriptional and posttranscriptional levels. Moreover, studies of preadipocyte cell lines have shown that genes inhibiting the adipogenesis process are repressed during the early and late stages of adipocyte differentiation [6].

## 2. Vitamin A

Vitamin A is a fat soluble vitamin found in both animal (retinol and its close derivatives) and vegetal (provitamin A; carotenoids) origin have an unsaturated isoprenoid chain structure. Carotenoids can be eventually converted to retinol; however, some of carotenoids cannot metabolized into retinoids. *β*-Carotene, *α*-carotene, lutein, lycopene, and cryptoxanthin are the most common carotenoids [7]. Epidemiological studies reported a correlation between carotenoids and cancer prevention because it acts as antioxidants that prevent deoxyribonucleic acid (DNA) damage by fighting the free-radicals [8].

Retinoids include natural and synthetic compounds with a general structure of four isoprenoid units. These compounds are liposoluble and are stored in the liver and adipose tissues and other parts of the body [9]. The main bioactive forms are 11-*cis*-retinal and all-*trans*-retinoid acid (ATRA) [10]. 11-*Cis*-retinal produced by the oxidation of retinol is essential for vision; ATRA, a transcription factor ligand regulate the expression of genes involved in cell morphogenesis, differentiation, and proliferation [11, 12].

Retinoic acid (RA) regulates the transcription factor activity of several members of the nuclear receptor family, including the classical RA receptors (RAR*α*, RAR*β*, and RAR*γ*) and peroxisome proliferator-activated receptor beta/delta (PPAR*β*/*δ*) [13]. Binding of RA to its receptors mediated by two intracellular lipid-binding proteins: cellular RA binding protein II (CRABP-II), which facilitates RA binding to RAR, and fatty acid binding protein type 5 (FABP5), which delivers RA to PPAR*β*/*δ* [14, 15]. Moreover, RA and ATRA induce PPAR*β* and PPAR*δ* expression by acting as ligands for retinoid X receptor (RXR) [16].

Retinol binding protein 4 (RBP4) is a novel adipocytokine that may link obesity and insulin resistance. RBP, encoded by the RBP4 gene is a transporter protein deliver retinol from blood to extrahepatic tissue. RBP4 originates from the liver, and some other tissues, such as lung, adipose tissue, kidney, brain, and epithelial cells in the eye [17]. It is a 21 kDa adipokine specifically expressed in mature adipocytes. In addition, increased secretion of adipokines and cytokines lead to high level of proinflammatory, such as C-reactive protein (CRP) [18], tumor necrosis factor alpha (TNF*α*) [19], interleukin 6 (IL6) [20], and interleukin 34 (IL34) [21, 22].

RBP4 is a unique adipocytokine that seem to be link obesity and insulin resistance. High mobility group A1 gene (HMGA1) was identified early as an inducer of RBP4 in both human and mice [23]. In addition, the transcription of RBP4 is regulating by HMGA1 through cyclic adenosine monophosphate (cAMP) pathway [24].

Besides the adipokines and cytokines roles in the induction of insulin resistance, it has been found that elevated level of RBP4 may linked to obesity, and other risk factor, such as cardiovascular disease, hypertension, and hyperlipidemia [25, 26]. Higher serum concentration of RBP was shown correlated with higher level of glucose, triglycerides, and cholesterol in human [27, 28].

It was shown that obese and diabetic mice and human have a high level of RBP4 level in adipose tissue. Moreover, overexpression of RBP4 led to decrease glucose intolerance and increase insulin resistance in mice. On the contrary, deletion of RBP4 in mice showed improved insulin sensitivity [29]. Another study positively correlated obesity and type II diabetes mellitus with the inhibition of insulin signalling and upregulation of RBP4 [30]. However, low expression of RBP4 and other adipogenic genes is thought to be associated with DNA methylation at genes regulating adipocytokine signalling and insulin sensitivity pathways [31].

Studies provided different mechanisms for how RBP4 correlates with insulin sensitivity. One of them, show that hyperglycemia induced by high expression of RBP4 led to elevated phosphoenolpyruvate carboxykinase in the liver; and, thus, impaired insulin signalling in muscle [29]. The retinol RBP4 complex was also shown to obstruct insulin signalling in collaboration with STRA6 activates tyrosine phosphorylation through activation of Janus kinase 2 (JAK2) and signal transducer and activator of transcription 5 (STAT5) results in the induction of PPAR*γ* expression, which enhance adipocytes differentiation, and suppression of cytokine signalling 3 (SOCS3), which is well known as insulin signalling inhibitor [32].

Despite the fact of RBP4 impaired insulin sensitivity; however, studies shown that RBP4^−/−^ mice are indistinguishable from their wild-type in term to insulin sensitivity [33]. Moreover, acute and long-term overexpression of liver-specific RBP4 in murine increase circulating RBP4 levels to the same level observed in glucose intolerance and insulin resistance conditions; however, it did not disturb the glucose homeostasis suggesting that liver-secreted RBP4 is not associated with the development of glucose intolerance and insulin resistance [34]. In addition, transgenic overexpression of human adipocyte-specific RBP4 in mice was not affect RBP4 circulation level. However, transgenic mice were less sensitive to insulin and exhibited high concentrations of free fatty acid and triglyceride in serum [35] supporting a direct correlation between RBP4 and lipolysis [36]. Somewhat consistent is the finding that in vitro co-cultured of human adipocytes with macrophages promoted unesterified fatty acid synthesis by stimulates the secretion of pro-inflammatory cytokines that have been shown to play a potential role in adipose tissue lipolysis through controlling insulin-signalling pathway [37].

Preclinical studies conducted were conducted on obese mice to evaluate the effect of administration of vitamin A on obesity and vitamin A signalling in liver. They found that vitamin A level markedly reduced in several organs including liver in obese mice. Moreover, the transcription signalling of vitamin A was impaired and that including suppression of RA receptor mRNAs and reduction of cellular retinol binding protein 1 (CRBP1) in liver associated with the increase in adiposity and fatty liver [38].

Another study compared the influence of feeding “Spargue-Dawley rats” sufficient or deficient vitamin A diet for 8 weeks on fat mass documented that sufficient vitamin A diet was able to reduce the epididymal fat mass [39]. Vitamin A deficient diet feeding resulted in a marked increase in total fat content and decrease of PPAR*γ*2 expression in vivo. Indeed, treatment of mice with ATRA led to considerable reduction on body weight and adiposity [40].

## 3. Genetic Programming in Adipogenesis: PPARs and C/EBPs Factors

PPAR*γ* is a transcription factor and member of the nuclear receptor superfamily that heterodimerises with the RAR and binds to its response elements in the target gene's promoter region to induce transcription [41].

Stem cell precursors differentiate into mesenchymal lineages, which can differentiate into other mesenchymal lineages, including adipocytes, under appropriate treatment. Pre-adipose cell line differentiation can be stimulated by the glucocorticoid receptor and the cAMP-dependent protein kinase pathway [42]. These signalling molecules alter morphology and induce gene expression in mature adipocytes [2, 43, 44].

The PPAR nuclear receptor superfamily comprises three different subtypes (*α*, *β*/*δ*, and *γ*), all of which have important regulatory roles in lipid and glucose metabolism in several tissues types, including skeletal muscle, liver, and adipose [45]. Each PPAR subtype has a specific tissue distribution and ligand specificity [46]. PPAR*α* is a ligand-activated nuclear receptor that is highly expressed in the liver, heart, macrophages, and intestine. It is activated by saturated and unsaturated fatty acids, leukotriene B4, and 8-hydroxyeicosatetraenoic acid [47].

PPAR*β*/*δ* is expressed in tissues that express considerable amounts of mRNA, including the brain, skin, liver, skeletal, and adipose [48]. Moreover, RA has been identified as a physiological ligand of the PPAR*β*/*δ* nuclear receptor, controlling cell survival. PPAR*β*/*δ* expression is stimulated by 4-hydroxynonenal and prostacyclin [49].

PPAR*γ* is a key regulator of adipocyte development in vitro and in vivo and is sufficient for the transdifferentiation of nonadipocytes into adipocyte-like cells [50, 51]. PPAR*γ* is expressed in white and brown adipose tissues, the placenta, the large intestine, and macrophages. It has three promoters, PPAR*γ*1, PPAR*γ*2, and PPAR*γ*3; PPAR*γ*1 is active in various tissues, PPAR*γ*2 is active only in adipose tissue, and PPAR*γ*3 is highly active in macrophages, the large intestine, and white adipose tissue [52]. Several physiological substances act as PPAR*γ* ligands, including unsaturated fatty acids, 15-hydroxyeicosatetraenoic acid, and flavonoids [53].

CCAAT-enhancer-binding protein (C/EBP) transcription factors also regulate adipogenesis. There are six members of the C/EBP basic leucine zipper family, and three (*α*, *β*, and *δ*) have important roles in adipocyte differentiation. C/EBP*α* exists in two isoforms, p30 and p42, with the latter considered the most potent transactivator [54]. Studies on the three C/EBP*β* isoforms (liver-enriched activating protein [LAP], LAP∗, and liver-enriched inhibiting protein [LIP]) have found LAP and LAP∗ to be potent transactivators; however, LIP is not a potent transactivator because it is missing the transactivation domains present in LAP and LAP∗ [55]. Suppression of C/EBP*β* and C/EBP*δ* in mice reduced the size of adipose tissue pads [56]. Interestingly, preadipocytes required C/EBP*β* for MCE [57] and C/EBP*α* for proper white adipocyte differentiation. In addition, C/EBP*α* expression was stimulated after MCE due to its antimitotic activity [58]. C/EBP*β* and C/EBP*δ* play an essential role in terminal differentiation of brown, but not white adipocytes in vivo [59].

The in vitro initiation of adipocyte differentiation requires induction of C/EBP*β* and C/EBP*δ*, which in turn induce PPAR*γ* and C/EBP*α* expression, resulting in the activation of adipocyte-specific genes. There is evidence suggesting that RA can inhibit adipogenesis by blocking C/EBP*α* and PPAR*γ* expression [60]. However, RA loses its inhibitory effects in the presence of C/EBP*α*. Furthermore, PPAR*γ* and C/EBP*α* can induce each other's expression, promoting, and maintaining the mature adipocyte phenotype [2].

Mutation or deletion of the PPAR*γ* gene in white adipocytes completely inhibits adipogenesis and causes cardiovascular diseases, fatty liver, and insulin-resistant diabetes [61, 62]. In contrast, cells lacking C/EBP*α* can differentiate into adipocytes, although these differentiated cells have fewer accumulated lipid droplets and do not express PPAR*γ*. Therefore, coregulation of PPAR*γ* and C/EBP*α* is important for maintaining normal differentiation. Moreover, differentiating adipocytes express several transcription factors interacting at different stages of adipogenesis to produce mature adipocytes.

Other transcription factors involved in adipocyte differentiation include early growth response 2, Krüppel-like factors (KLF) [63], sterol-regulatory element-binding protein (SREBP) 1C [64], and STAT5 [65]. However, it appears that their function depends on the regulation of C/EBP*β*, C/EBP*δ*, C/EBP*α*, and PPAR*γ* activity. Moreover, while PPAR*γ* induced adipogenesis in C/EBP*α*^−/−^ mouse embryonic fibroblasts (MEFs), C/EBP*α* did not affect adipogenesis in PPAR*γ*^−/−^ MEFs [66].

## 4. Retinoic Acid and Adipogenesis

Retinoids are potent inhibitors of adipocyte differentiation depending on the differentiation stage, RA concentration, and retinoid receptor availability in adipocytes. Vitamin A metabolite RA strongly inhibits adipogenesis in cultured cells in early stages of differentiation [67, 68]. More recent evidence has shown that the inhibitory effect of RA wanes as differentiation progresses due to RAR inhibition following differentiation induction [69]. Moreover, adipocyte differentiation is accompanied by CRABP-II downregulation and PPAR*β*/*δ* and FABP5 upregulation. In addition, the hormone signals of RA act through both the CRABP-II and RAR pathways in preadipocytes [70]. However, it remains unclear whether RA directly affects marker genes expressed in mature white adipocytes.

Adipose tissue proliferation depends on hyperplasia (increased adipocyte size) or hypertrophy (increased cell number). ATRA inhibits the expression of C/EBP*α*, PPAR*γ*, and their target genes. ATRA-induced stimulation of RAR expression inhibited RAR*γ*, but not RAR*α* expression and attenuated ATRA-induced reduction of PPAR*γ*2 expression [71].

**Figure 1:**
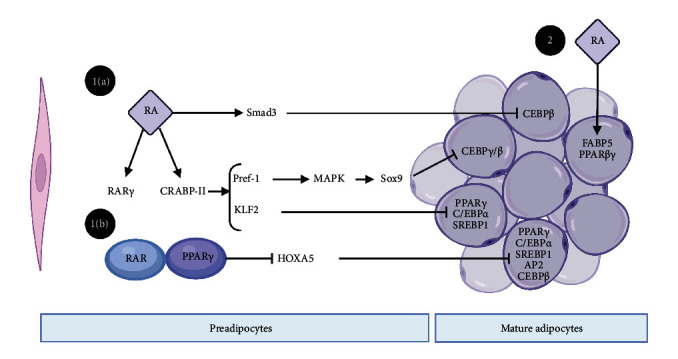
RA-mediated regulation of adipocyte biology. RA regulates adipose tissue biology via two distinct pathways. (a) In preadipocytes, RA activates CRABP-II and RAR to inhibit adipocyte differentiation and upregulates PREF-1, SMAD3, and KLF2, inhibiting C/EBPs and PPAR*γ* expression, and adipocyte differentiation. (b) In mature adipocytes, RA-induced activation of the PPAR*γ*/RXR heterodimer inhibits the expression of HOXA5 and audiogenic genes and activates the CRABP-II/RAR and FABP5/PPAR*β*/*γ* pathways to promote lipid oxidation and energy utilisation.

RA induced the expression of preadipocyte hallmark markers preadipocyte factor 1 (PREF-1), KLF2, and sex-determining region Y box 9 (SOX9) by activating CRABP-II and RAR*γ* (Figure 1). Therefore, RA inhibits adipocyte differentiation and maintains preadipocyte morphology and phenotype. In contrast, RA regulates gene expression that enhances lipid oxidation, insulin response, and energy expenditure in mature adipocytes by activating RAR and RXR. RA prevented adipocyte hypertrophy in mice by inhibiting the formation of new adipocytes and stimulating energy expenditure [72].

RA-mediated inhibition of preadipocyte differentiation results from RAR interference by C/EBP*β* that is initiated by SMAD3. It has been previously reported that RA induces Pref-1 expression, an alternative pathway for inhibiting C/EBP*β* expression by triggering extracellular signal-regulated kinase (ERK)/mitogen-activated protein kinase (MAPK) signalling, inducing SOX9 expression. SOX9 is a known inhibitor of C/EBP*β* and C/EBP*γ* expression. Pref-1 overexpression in transgenic mice led to partial lipodystrophy and hypertriglyceridemia. However, lipodystrophy was not accompanied by RA-mediated induction of Pref-1. KLF2 suppresses adipocyte differentiation by inhibiting PPAR*γ*, C/EBP*α*, and SREBP1C expression by targeting RAR and CRABP-II in preadipocytes. KLF2 promotes RA signalling through CRABP-II and RAR*γ*, enhancing the inhibition of adipogenesis (Figure 1).

## 5. HOX

Homeobox (HOX) is a 180-bp DNA sequence encoding a 60-amino acid DNA-binding domain (homeodomain). It is believed that proteins containing a homeodomain are transcription factors with key roles in the anatomical development (morphogenesis) of various organisms. Moreover, several homeodomain proteins regulate apoptosis, the cell cycle, cancer, and many differentiation programs, including adipocyte differentiation, by stimulating the expression of target genes required for specific tissue and organ formation [73]. Human HOX genes are found in four distinct clusters: HOXA, HOXB, HOXC, and HOXD [74]. Evidence shows that HOX gene expression differs in human subcutaneous and abdominal fat [73].

Studies have identified HOX gene involvement in adipocyte differentiation that is regulated by PPAR*γ* [75]. A paralogous group of seven genes (*HOXA5*, *HOXA1*, *HOXB4*, *HOXD9*, *HOXD8*, *HOXD4*, and *HOXD3*) is expressed only in non-differentiated adipocyte cells, whereas *HOXA5*, *HOXA10*, *HOXB9*, and *HOXD10* are highly expressed during early differentiation, *HOXA4*, *HOXA7*, and *HOXD4* are expressed only in differentiated adipocytes [76, 77]. Their expression level depends on the localisation of fat depots in the human body [77]. The transcriptional roles of PPAR*γ* during adipocyte differentiation and RA in human HOX gene induction have been confirmed [76].

HOX genes are coactivated by a heterodimeric RAR/RXR complex. RAR forms a heterodimer with RXR to induce HOX gene expression in the presence of a histone deacetylase (HDAC) inhibitor. Without the HDAC inhibitor, RXR heterodimerises with PPAR*γ* to suppress HOX gene expression (Figure 1). HOXA5 is epigenetically regulated, and its expression is regulated in a time-dependent manner [78, 79].

HOXA5 expression is enhanced by RA [80], regulating adipocyte differentiation and body fat accumulation [81]. HOXA5 overexpression significantly increased PPAR*γ*, CEBP/*α*, CEBP/*β*, apetala 2, and SREBP1 expression in goat subcutaneous preadipocytes. HOXA5 downregulation notably decreased the accumulation of lipid droplets and adipogenic gene expression [82].

Treating mice with an RXR and PPAR*γ* antagonist depleted white adipose tissue and markedly decreased the triglyceride content of white adipose tissue, protecting against obesity and related diseases, such as type 2 diabetes [83].

HOXA5 expression is high in preadipocytes and declines during adipogenesis. There is a strong correlation between adipocyte differentiation, mitochondrial biogenesis, and HOXA5 expression in vitro. However, the regulatory mechanisms of HOXA5 on adipocyte differentiation and mitochondrial biogenesis remain unknown. Moreover, C/EBP*β* binding to the HOXA5 promoter strongly suppressed its methylation [81, 84].

## 6. PREF-1 and SOX9

Delta-like non-canonical notch ligand 1 (DLK1/PREF-1) is a plasma membrane protein expressed exclusively in preadipocytes. It is a potent inhibitor of differentiation through activation of ERK/ MAPK, inducing SOX9 expression, which inhibits the C/EBP*β* and C/EBP*δ* expression and suppresses adipogenesis [85] [72, 86, 87]. In vivo mutation of the *Pref-1* gene increases differentiation and adipocyte marker gene expression. Pref-1 overexpression in mice appears to impair adipogenesis. Notably, the role of Pref-1 in adipocyte differentiation differs by cell type; Pref-1 overexpression activated adipogenesis in C3H10T1/2 mouse cells [88], but not in Pref-1-knockout mice, where it accelerated preadipocyte differentiation and increased adipocyte marker gene expression [89, 90]. However, Pref-1 overexpression in transgenic mice caused the formation of new fat cells and decreased the expression of mature adipocytes marker genes [87, 88, 91].

Similar to PREF-1, SOX9 is a transcription factor that negatively regulates early adipogenic differentiation and is downregulated by adipogenic signals. SOX9 inhibition is required for adipocyte differentiation [92].

## 7. KLF

KLF15 is a member of the KLF transcription factor family that is expressed only in mature adipocytes, inducing the expression of solute carrier family 2 member 4 (SLC2A4/GLUT4). A second KLF family member, KLF2/lung KLF, is a negative regulator of adipocyte differentiation that maintains the preadipocyte state by inhibiting PPAR*γ*, C/EBP*α*, and SREBP1C [93, 94]. Previous studies have shown that KLF2 is highly expressed in white and brown adipocytes, 3T3-L1 cells, and primary human preadipocytes; however, its expression is markedly decreased after adipocyte differentiation. Moreover, KLF2 overexpression in 3T3-L1 cells reduces intracellular lipid accumulation and decreases PPAR*γ*, adducin 1, SREBP1C, and C/EBP*α* expression, but not C/EBP*β* or C/EBP*δ* expression. In contrast, KLF15 does not affect PPAR*γ* expression [93].

KLF2 overexpression in preadipocytes prior to RA-induced differentiation led to marked inhibition of adipogenesis and upregulation of cellular retinol-binding protein II (CRABP-II) and RAR*γ*, confirming the involvement of KLF2 in RA-induced adipogenesis inhibition. Conversely, decreased KLF2 expression induced adipocyte differentiation and reduced the ability of RA to inhibit preadipocyte differentiation in NIH3T3-L1 and C3H10T1/2 cells [95].

## 8. SMAD

RA regulates SMAD family member 3 (SMAD3), which is involved in adipogenesis [96]. While the mechanism by which RA regulates Smad3 expression is unclear, some evidence suggests that it upregulates transforming growth factor *β*-effector protein Smad3 expression, which interacts with C/EBP*β* via its Mad homology 1 domain, interfering with C/EBP*β* DNA binding. Interestingly, RA alone is insufficient to inhibit adipocyte differentiation; it requires Smad3 to inhibit adipocyte differentiation and suppress C/EBP*β* expression [97]. In addition, RA-mediated stimulation of Smad3 expression increases both cytoplasmic and nuclear SMAD3 levels. Inducing Smad3 in preadipocytes without RA was insufficient to inhibit adipogenesis and C/EBP*β* expression. However, the inhibitory mechanism of RA on adipocyte differentiation is limited in the absence of Smad3, suggesting that Smad3 is an important mediator of the inhibitory effects of RA during adipogenesis [96].

## 9. Clinical Perspectives

Excess fat accumulation induces endoplasmatic reticulum stress in adipocytes and stimulates adipocytes to release free fatty acids and inflammatory mediators, such as necrosis factor alpha, IL6, and CRP as well as decrease adiponectin synthesis. As a result, increase oxidative stress and pro-inflammatory state [20, 98].

Evidence suggested that long-term low grade inflammatory basis led to obesity and associated co-morbidities [99, 100]. However, low and high dosage administration of vitamin A decrease TNF-*α* and IL6 on obese adults [101]; therefore, reducing insulin resistance, and improving energy expenditure [102].

Moreover, positive correlation has been revealed between T helper 17 (Th17) cells pools and the production of interleukin 17 (IL17). The Th17 and IL17 play detrimental roles in obesity. Interestingly, serum IL17 is upregulated in obese human patients [103]. However, vitamin A supplementation diminishes serum levels of IL17 and transforming growth factor beta (TGF-*β*) in premenopausal women [104]. Moreover, daily intake of vitamin A (25,000 IU/day) as retinyl palmitate increase circulating ATRA and 9-*cis* RA and reduce Th17 cells activity [105].

The association between serum RBP4 levels and resistance to inhibition of lipolysis by insulin is likely a cause-and-effect relationship in obese human subjects. In parallel, in cultured human adipocytes, RBP4 significantly activated lipolysis; however, it did not alter insulin suppression of lipolysis [37]. Although, not all studies agreed with the fact that circulating retinol concentrations differ in obese and non-obese adults; however, it has been confirmed that retinol:RBP4 inversely related to body mass index (BMI). Moreover, fasting glucose and insulin serum concentrations slightly lower in obese compared with normal subjects [106]. In addition, morbidly obese have higher fasting serum concentration of RBP4 compared with nonobese; however, RBP4 declined in obese subjects after gastric banding surgery [107].

Adverse relationship between high visceral and body adiposity and vitamin A nutritional status was observed in adult women [108]. Furthermore, greater expression of RBP4 mRNA in the visceral and subcutaneous adipose tissue was found in obese compared with non-obese adults [30]. Likewise, serum RBP4 positively correlated with visceral fat mass and treatment with PPAR*γ* agonist diminish RBP4 and visceral fat mass [109].

On other hand, strong inverse correlations between BMI and circulating *β*-carotene, *β*-cryptoxanthin, retinol, and other carotenoids were found [110]. Serum concentrations of retinyl ester and *β*-carotene seems to be inversely related obesity and metabolic syndrome in school-age children. Therefore, carotenoids supplementation may result in waist circumference and body weight losses [111, 112]. However, long-term treatment with high oral dose concentration of vitamin A (25,000 IU/day as retinyl palmitate) elevated serum lipids, liver enzymes, and CRP in obese and lean women [113]. Studies recommended oral administration of carotenoids to reduce the abdominal fat area including visceral and subcutaneous fats. Intake of 9 mg carotenoids for 12 weeks significantly reduced BMI without notable side effects [114]. Moreover, consumption of a beverage containing 2 mg *β* cryptoxanthin decrease the BMI and visceral fat in pre-obese men [115].

## 10. Conclusion

Data indicate that RA regulated adipose tissue biology via two mechanisms: inhibition of adipogenesis via activation of CRABP-II and RAR in preadipocytes and activation of the CRABP-II/RAR and FABP5/PPAR*β*/*γ* pathways in mature adipocytes to control energy utilisation and lipid peroxidation. Therefore, RA suppresses dietary-induced obesity by suppressing lipid accumulation and adipocyte hypertrophy. Thus, a better understanding for the role of RA in mediating the proliferation status genes expression may provide a therapeutic option for reversing adverse programming of obesity in humans.

Although, the well-established the role of RA in blocking adipocyte differentiation, enhancing weight losses in vitamin A rich diet fed rodents, and insulin resistance related metabolic disorder including BMI and obesity in human suggested alternative therapeutic options to use vitamin A to treat and prevent obesity. However, it is still not clear whether the cell culture and animal model data can be translated to human therapies.

## Data Availability

There are no raw data associated with this review article.

## Abbreviations

PDGFR: Platelet-derived growth factor receptor
MCE: Mitotic clonal expansion
mRNA: messenger RNA
ATRA: All-trans-retinoid acid
RA: Retinoic acid
MCE: Mitotic clonal expansion
FABP5: Fatty acid binding protein type 5
RBP4: Retinol binding protein 4
TNF*α*: Tumor necrosis factor alpha
IL6: Interleukin 6
IL34: Interleukin 34
HMGA1: High mobility group A1 gene
cAMP: Cyclic adenosine monophosphate
JAK2: Janus kinase 2
STAT5: Signal transducer and activator of transcription 5
PPAR*γ*: Peroxisome proliferator-activated receptor gamma
SOCS3: Suppression of cytokine signalling 3
CRBP1: Cellular retinol binding protein 1
RAR: Retinoic acid receptor
C/EBP: CCAAT-enhancer-binding protein
LAP: Liver-enriched activating protein
LIP: Liver-enriched inhibiting protein
KLF: Krüppel-like factors
SREBP: Sterol-regulatory element-binding protein
MEFs: Mouse embryonic fibroblasts
PREF-1: Preadipocyte factor 1
SOX9: Sex-determining region Y box 9
HOX: Homeobox
DLK1/PREF-1: Delta-like non-canonical notch ligand 1
ERK: Extracellular signal-regulated kinase
MAPK: Mitogen-activated protein kinase
SMAD3: SMAD family member 3
CRP: C-reactive protein
Th17: T helper 17
IL17: Interleukin 17
TGF-*β*: Transforming growth factor beta
BMI: Body mass index.
